# Central conduction time in auditory brainstem response and ear advantage in dichotic listening across menstrual cycle

**DOI:** 10.1371/journal.pone.0187672

**Published:** 2017-11-09

**Authors:** Xu-Jun Hu, Chi-Chuen Lau

**Affiliations:** 1 College of Medical Technology, Zhejiang Chinese Medical University, Hangzhou, China; 2 Independent Scholar, Des Plaines, Illinois, United States of America; University of New England, Australia, AUSTRALIA

## Abstract

The ovarian hormones fluctuate during the menstrual cycle in women. Such fluctuation of sex hormones, in particular estrogen, is believed to affect the central conduction time in auditory function as well as the language lateralization in cognitive function. However, findings are inconsistent. The underlying mechanisms are also unclear. This paper examined if there was any relation between the central conduction time and the language lateralization at different times during the menstrual cycle. Twenty young women with normal menstrual cycle were tested four times (5 to 7 days apart) across the menstrual cycle. The test battery included the electrophysiological measurement of auditory evoked response in brainstem and the speech performance in dichotic listening with monosyllables as stimulus pairs. The dichotic listening task was conducted under the non-forced, forced-right and forced-left attention. The central conduction time was defined by the time elapsed between two auditory elicited responses along the auditory pathway. The language lateralization in dichotic listening was expressed in ear advantage, which was the right-ear score minus the left-ear score. The results showed that the effects of test time were significant on both the central conduction time and the ear advantage under the forced-left attention. Overall, the interaural difference in the central conduction time correlates with the ear advantage (non-forced attention) at the beginning of the menstrual cycle. The change in central conduction time between two test times correlates significantly with the change in ear advantage under the non-forced and forced-left attention. Conclusively, the central conduction time depends on the time during the menstrual cycle, which in turn may affect the performance in dichotic listening.

## Introduction

The sex hormones in females, primarily estrogen and progesterone, fluctuate during the menstrual cycle [1]. The changes in sex hormones have been shown to influence the neurophysiological processing of auditory information as recorded in the auditory brainstem response [2] and the language lateralization as measured in the dichotic listening task [3]. However, there is limited data available to examine any relation between the findings in these two research paradigms.

The auditory brainstem response (ABR) is a tool commonly used to examine the effect of the menstrual cycle on auditory function [4]. ABR is a far-field electrical recording of the neuron activities in brainstem during the processing of sound transmitted from an acoustic transducer such as an insert earphone. When the acoustic signal travels along the auditory pathway from the cochlear nuclear complex to the inferior colliculus, it elicits a series of neuron activities at certain sites. The evoked neuron activities can be measured electrically as peaks and troughs by the surface electrodes typically placed at the vertex of the scalp and the ear lobes. The peaks of the evoked potential on the electrical waveform are labeled I to V in roman numerals, of which the peaks I, III and V are more reliable. Peak I is the action potential generated at the peripheral portion of the eighth cranial nerve in the cochlea. Peak III is the postsynaptic action potential elicited at the ventral cochlear nucleus, while peak V is resulted from the postsynaptic action at the lateral lemniscus. Two measurements are typically used in research: the time elapsed (i.e., wave latency) for a peak action potential to occur and the interpeak conduction time for the signal to travel between two major neuron sites [5]. The peaks in the waveform normally occur within the first 10 ms after a click or tone-burst stimulus has been presented at 70–90 dB nHL.

Although studying the effects of fluctuating sex hormones on ABR seems straightforward, the findings as reported in women with normal menstruation cycle are inconsistent and conflicting. The results can range from no significant difference across the menstrual cycle [6] to a significant increase in the wave latency at the follicular phase but not the interpeak conduction time [7]; and from a significant increase in the wave latency and the interpeak conduction time at the follicular phase [4–5, 8–10] to an opposite outcome [11]. Therefore, the impacts of fluctuating sex hormones on auditory function as measured with ABR are still uncertain.

On the other hand, the language lateralization measured with dichotic listening is a functional approach to examine the effects of fluctuating sex hormones on the performance of a specific cognitive task [12]. As the human cortex is asymmetric in cognitive function, there is a differential involvement of the left and the right hemispheres in performing the cognitive task. For example, the left hemisphere is mostly responsible for processing language for healthy adults. Commonly, the dichotic listening task is employed to study the language lateralization [13–15]. This test requires a listener to report the word (or nonsense syllable) heard in each ear after two stimuli have been presented simultaneously but separately to the left and the right ears. The dichotic listening task usually shows a bias toward the right ear, resulting in a right-ear advantage (REA), which is more prominent for the right-handed listeners [16]. The anatomy of the brain may explain the REA in dichotic listening. A speech signal delivered to the right ear travels a direct, contralateral pathway to the speech processing areas at the left hemisphere. The impulses from the right ear traveling along this contralateral (primary) pathway can partially suppress the impulses from the left ear along the ipsilateral (secondary) pathway at the overlap of the two pathways, and this slight right-ear advantage can be further intensified by the central competition [13]. Moreover, the impulses from the left ear travelling (contralaterally) to the right hemisphere have to take a longer time to cross the corpus callosum before reaching the speech processing areas at the left hemisphere [17]. This delay for the signal from the left ear to arrive at the speech processing areas also contributes to the right-ear advantage [12].

Interestingly, the reports on the effects of fluctuating sex hormones on language lateralization measured with dichotic listening are controversial too. The findings can range from an increased REA when the levels of estrogen and/or progesterone are high [18–20] to a reduced REA at the luteal phase [21–22], and to no significant change in REA [2, 12, 23–24].

Furthermore, the effects of fluctuating sex hormones on language lateralization can be examined when listeners are instructed to pay attention to sounds heard in either the left or the right ear. Hjelmervik et al. [2] reported a significant increase in the left-ear advantage at the follicular phase during the forced-left attention condition. However, there are other reports showing the language lateralization is independent of the attention condition [12,25].

The conflicting results in the effects of the menstrual cycle on the central conduction time in ABR and the ear advantage in dichotic listening may be due to the differences in research designs. For example, the data in ABR can be obtained with each ear tested separately [5] or with both ears stimulated simultaneously [9]. In dichotic listening, using real words as stimuli probably requires more working memory to process than using nonsense syllables [12]. The method to define the menstrual cycle-phases (i.e., the test days) can also be a factor to influence the findings. The method of day count is typically based on a self-reported menstrual history to define the cycle-phases. However, the method of hormone assays is used to classify the cycle-phases with more precision [25]. The methods to capture ovulation phase are also different, including the use of basal body temperature [26] and the ovulation home detection kit [27].

Hodgetts et al. [25] summarized ten studies on the change of ear advantage in dichotic listening across the menstrual cycle. The five studies that used the method of daycount showed a reduced right-ear advantage at either the menstrual phase (3–5 days after menses) or the luteal phase (21–28 days). For the other five studies that used hormone assays, one reported no menstrual cycle effect but the other four reported similar results as the studies with the method of day count. Apparently, both methods, depending on the nature of the study, are acceptable for investigating the effects of fluctuating sex hormones on dichotic listening.

As the dichotic listening task depends on the understanding of signals that have traveled along the auditory pathways to the speech processing areas, the central conduction speed of the signals may be a factor influencing the speech performance in each ear. The central conduction speed is inversely proportional to the central conduction time that can be measured in ABR. Therefore, the current paper was designed to examine the interactions between the central conduction time and the ear advantage in dichotic listening (i.e., difference in performance between two ears) at different times across the menstrual cycle. The hypothesis was that the menstrual cycle at different times could affect the central conduction time differently, which in turn influenced the performance in dichotic listening. Specifically, the goal was to answer three questions: (1) Does the central conduction time depend on the time of measurement across the menstrual cycle? (2) Does the ear advantage in dichotic listening depend on the time across the menstrual cycle? (3) Is there any relation between the central conduction time and the ear advantage in dichotic listening at different times during the menstrual cycle?

## Method

### Participants

To determine an appropriate sample size for the current study, reference was made to Battaet al. [10] who reported significant difference in wave latency at peak V across the menstrual cycle, and to Hjelmervik et al. [2] who reported significant difference in ear advantage under the forced-left attention. Apower analysis software (G*Power v3.1.9.2, Heinrich Heine University, Germany) was used to estimate the sample size for the power to reach 0.8. Based on these two studies [2, 10], a sample size larger than 15 was required to examine the effects of the menstrual cycle on measurements in ABR and dichotic listening simultaneously.

Twenty (20) female college students with a mean age of 21.5±0.8 years participated in the current study. They could repeat correctly all the English speech stimuli presented monaurally. They were healthy with a mean BMI 18.20±0.94 kg/m^2^. Their first menstrual cycle occurred at age 13.0±1.7 years. The length of menstrual cycle for each participant was monitored for 3 months with a menstruation program (Dayima App v.5.0, Beijing, China) before the actual test. The participants were instructed to record the beginning of each menstrual cycle and the basal body temperature daily. The basal body temperature was used to verify ovulation for each participant [9]. Based on the data in the menstruation program, all participants had a regular, natural menstrual cycle of length 26 to 31 days. During the candidacy selection, several known factors that could interact with the effects of estrogen on auditory function and/or cognitive function were excluded. The participants had no history of abortion, pregnancy and use of contraceptives during the last 6 months prior to the study [2, 5]. They also had no history of mental and neurological illnesses, tinnitus, central auditory processing disorder [5], ototoxic drug [28] and noise exposure [29]. Their otoscopic examination was normal and their hearing thresholds measured with an audiometer (AC40, Interacoustics, Middelfart, Denmark) were better than 25 dBHL bilaterally between 250Hz and 8000Hz. Only right-handed participants, confirmed with the Edinburgh Handedness Inventory—short form [30] by a laterality quotient score > 61, were chosen to maximize the degree of left language lateralization in dichotic listening [16]. All participants signed a consent form and received no financial compensation for the study. The review board of Zhejiang Chinese Medical University, China, approved this research for experimentation with human subjects.

### Test battery

#### Auditory brainstem response

An ABR machine (Charter EP200, ICS, Schaumburg, USA) was used to measure the wave latencies of peaks I, III, and V. The stimuli presented to the test ear were clicks (broadband noise) adjusted to 80 dBnHL with a repetition rate of 19.3 cycles per second. The time window was 15 ms and the filtering was 0.1–3.0 kHz for averaging the responses with 2048 sweeps. The sampling rate was 20 kHz. During the ABR test, the scalp areas where the electrodes would be placed were rubbed with alcohol to ensure the electrode impedance < 5kΩ. The active electrode was placed at the high forehead, the reference electrode at the earlobe of the test ear, and the ground electrode at the low forehead. For each participant, the ABR test was performed for both ears.

#### Dichotic listening test

The dichotic listening paradigm is a closed-set test developed by Kimura [13]. The stimulus set is composed of six consonant-vowel nonsense syllables: /ba/, /da/, /ga/, /ka/, /pa/, /ta/. Totally, there are 36 stimulus pairs (e.g., /ba/—/da/) that also include 6 homonymic pairs (e.g., /da/—/da/). The dichotic listening test with English stimuli in the iDichotic App (Bergen fMRI group, Bergen, Norway) can be conducted conveniently with a Smartphone [31–32]. To make the original iDichotic App to be usable by the local participants, all the test instructions needed to be in Chinese. To do this, all the original 36 stimulus pairs were first saved as wav files with a sound-analyzing program (Adobe Audition 3.0, San Jose, USA) installed in a Macintosh computer (MacBook, Cupertino, USA). The stimulus pairs were then randomized to generate 3 sets of test materials. Each set was used for one of the three attention conditions. An instruction in Chinese was added to each set of stimulus pairs accordingly. There was a 5-second interval between two stimulus pairs, giving listeners enough time to respond. All 3 sets of stimuli were then burned into a compact diskette (CD). In the non-forced attention, the listeners were instructed to report the syllables they heard most clearly in two ears. For the two attention-forced conditions, the listeners were instructed to pay attention to either the right ear or the left ear. During the actual test, the stimulus pairs were presented with the CD player in the audiometer. The listeners heard the stimulus pairs through the headphones (TDH39, Telephonics, New York, US). They wrote down on a response sheet the syllables they heard in each ear. The score (%) for each ear in a particular attention condition was the ratio between the correct responses and the total target syllables; the homonymic pairs were not counted toward the final score. The ear advantage (EA) is defined as the right-ear score minus the left-ear score. A positive EA indicates a right-ear advantage and a negative EA shows a left-ear advantage. The laterality index or quotient, defined as the ear advantage relative to the summed scores of both ears [2, 12, 25], is not reported in this paper.

#### Procedure

The participants were tested in a sound booth (A204S, Tuoenkang, China) with ambient noise less than 30 dB(A). All participants were tested four times across the menstrual cycle on the 3^rd^ or 4^th^ day after the beginning of menses (1^st^ time), the 9^th^ or 10^th^ day (2^nd^ time), the 14^th^ or 15^th^ day (3^rd^ time), and the 21^st^ or 22^nd^ day (4^th^ time). The test times in this study approximated the typical days in those studies [5, 19–21] that applied the day-count method to estimate the four cycle phases (menstrual, follicular, ovulatory, luteal). Since the four test times were not defined by the method of hormone assays, any findings would only demonstrate the overall effects of the menstrual cycle but not an assumed hormonal effect, as the exact levels of sex hormones for individual participants in each test time were not known.

In each test time, ABR and dichotic listening were done consecutively. Each session took approximately 2 hours including breaks between tests. This would ensure that both ABR and dichotic listening were under the influence of similar sex hormones. The start of the four test times was counterbalanced with four sequences: 1^st^-2^nd^-3^rd^-4^th^; 2^nd^-3^rd^-4^th^-1^st^; 3^rd^-4^th^-1^st^-2^nd^; 4^th^-1^st^-2^nd^-3^rd^. Therefore, except those tested with the sequence 1^st^-2^nd^-3^rd^-4^th^, the other participants were tested 4 times across two consecutive menstrual cycles. The order of measuring ABR and dichotic listening were also counterbalanced. During the dichotic listening test, all participants were tested first with the non-forced attention; the two attention-forced conditions were counterbalanced.

#### Statistical analysis

The statistical package SPSS 12.0 (IBM, Armonk, USA) was employed for data analysis. All datasets were first tested for normality with the Shapiro-Wilk procedure. For ABR, all 24 datasets of wave latency and interpeak conduction time (both ears) across the four test times were normally distributed. Moreover, all 24 datasets of ear score for dichotic listening were also normally distributed.

The global repeated-measures ANOVA (analysis of variance) was conducted to examine all the main effects on each independent variable in the ABR measurement and the dichotic listening task. For each main effect, the Mauchly’s test of sphericity was employed to examine if the variances of differences between all combinations of related levels are equal. When sphericity was violated, the Greenhouse-Geisser correction was applied. The F-value, significance level, effect size and the estimated power of test were calculated. The post-hoc analysis was performed with one-way repeated-measures ANOVA to investigate if the menstrual cycle (i.e., test time) had a significant effect on the independent variable. Multiple comparisons with paired-samples t test, adjusted with Bonferroni correction, were then conducted to identify which test time was significantly different from the other test times, *p*<0.0125. The relation between the measurements in the ABR test and the ear scores in dichotic listening was determined with the Pearson’s correlation coefficient.

## Results

### Effects of menstrual cycle on auditory brainstem response

The data analysis in ABR was performed for each ear separately and the average across ears for every participant. The analysis for each ear allows one to examine if the effect of menstrual cycle on each ear is different. The analysis with the average across ears is used to examine the combined effect of menstrual cycle on the hearing function [26].

**Fig 1:**
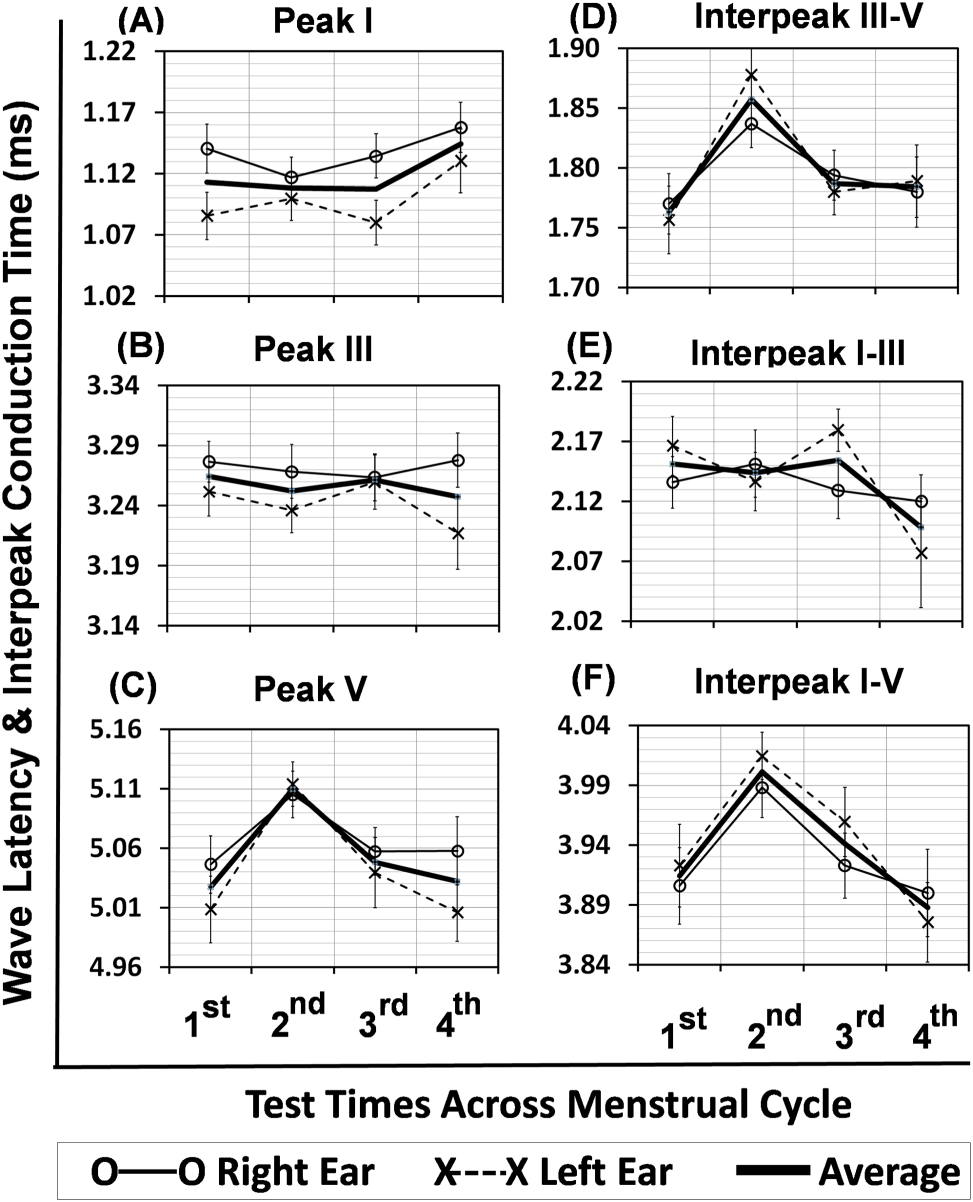
ABR wave latency and interpeak conduction time across the menstrual cycle. Error bars are standard errors.

For each test time, the wave latency for the peaks I, III and V and the interpeak conduction time were measured for the left and right ears separately. Fig 1 shows the mean wave latencies for the peaks I, III, and V (Fig 1A, 1B and 1C) and the conduction times for the interpeaks III-V, I-III, and I-V (Fig 1D, 1E and 1F) at four test times. The range along the vertical axis in each plot of Fig 1 is 0.20 ms. Overall, the patterns are similar between the right and left ears for both the peaks and the interpeaks across the menstrual cycle. The interaural difference at each test time is not greater than 0.06 ms. The wave latency for peak V is longest at the 2^nd^ test time, while the interpeak conduction time is also highest at this test time for the interpeaks III-V and I-V.

The 4x3 repeated-measures ANOVA was used to examine two main effects of test time (1^st^, 2^nd^, 3^rd^, 4^th^) x peak (I, III, V) on the wave latency, and also test time x interpeak (I-III, III-V, I-V) on the interpeak conduction time. Further analysis with one-way repeated-measures ANOVA was employed to examine the test times across the menstrual cycle. The results are shown in Table 1. The wave latency of peak V at the 2^nd^ test time is significantly longer than that at the 1^st^, 3^rd^ and 4^th^ test times for the left ear and the average across ears but not the right ear. The wave latency of other peaks is not changed across the test times. Consequently, the conduction time of the interpeak I-V at the 2^nd^ test time is also significantly longer than that at the other three test times for the average across ears, 1^st^ and 4^th^ test times for the right ear, and 4^th^ test time for the left ear. The conduction time for the interpeak III-V is only significantly longer at the 2^nd^ test time compared with the 1^st^ test time for the left ear and the average across ears.

**Table 1:** Results of analyses on wave latency and interpeak conduction time.

	4x3 ANOVA on wave latency					4x3 ANOVA on interpeak conduction time				
Ear	Effects	F	*p*	η^2^	power	Effects	F	*p*	η^2^	power
Right	Test time	0.51	0.67	0.02	0.14	Test time	7.15	<0.001	0.27	0.97
	Peak	18826	<0.001	0.99	1.00	Interpeak	5767.8	<0.001	0.99	1.00
	Interaction	2.37	0.06	0.11	0.66	Interaction	3.20	0.03	0.14	0.65
Left	Test time	3.17	0.03	0.14	0.70	Test time	7.15	<0.001	0.27	0.97
	Peak	21007	<0.001	0.99	1.00	Interpeak	5767.8	<0.001	0.99	1.00
	Interaction	4.72	<0.001	0.19	0.98	Interaction	3.20	0.03	0.14	0.65
Average	Test time	1.73	0.17	0.08	0.40	Test time	8.53	<0.001	0.30	0.99
	Peak	25928	<0.001	0.99	1.00	Interpeak	8524.4	<0.001	0.99	1.00
	Interaction	5.27	<0.001	0.21	0.96	Interaction	2.80	0.05	0.12	0.60
		Post-hoc: 1-way ANOVA					.			
Ear	Peak/Interpeak	Effect	F	*p*	η^2^	power	Significant difference among 4test times(1^st^, 2^nd^, 3^rd^, 4^th^)? ^a^			
Right	I	Test time	2.46	0.09	0.11	0.46	None			
	III		0.23	0.87	0.01	0.09	None			
	V		2.24	0.09	0.10	0.54	None			
	I-III	Test time	0.70	0.55	0.03	0.19	None			
	III-V		2.08	0.11	0.09	0.50	None			
	I-V		3.51	0.02	0.15	0.75	2^nd^> 1^st^, 4^th^			
Left	I	Test time	2.45	0.10	0.11	0.45	None			
	III		1.44	0.23	0.07	0.36	None			
	V		7.40	<0.001	0.28	0.97	2^nd^>1^st^, 3^rd^, 4^th^			
	I-III	Test time	3.29	0.05	0.14	0.58	None			
	III-V		4.48	0.006	0.19	0.86	2^nd^> 1^st^			
	I-V		6.84	0.001	0.26	0.97	2^nd^> 4^th^			
Average	I	Test time	3.65	0.04	0.16	0.60	None			
	III		0.42	0.73	0.02	0.12	None			
	V		6.57	<0.001	0.25	0.96	2^nd^>1^st^, 3^rd^, 4^th^			
	I-III	Test time	2.58	0.08	0.11	0.50	None			
	III-V		4.78	0.004	0.20	0.88	2^nd^>1^st^			
	I-V		8.16	<0.001	0.30	0.98	2^nd^>1^st^, 3^rd^, 4^th^			

Average: average across ears.

^a^ Multiple comparisons, significance level <0.0125.

### Effects of menstrual cycle on ear advantage

**Fig 2:**
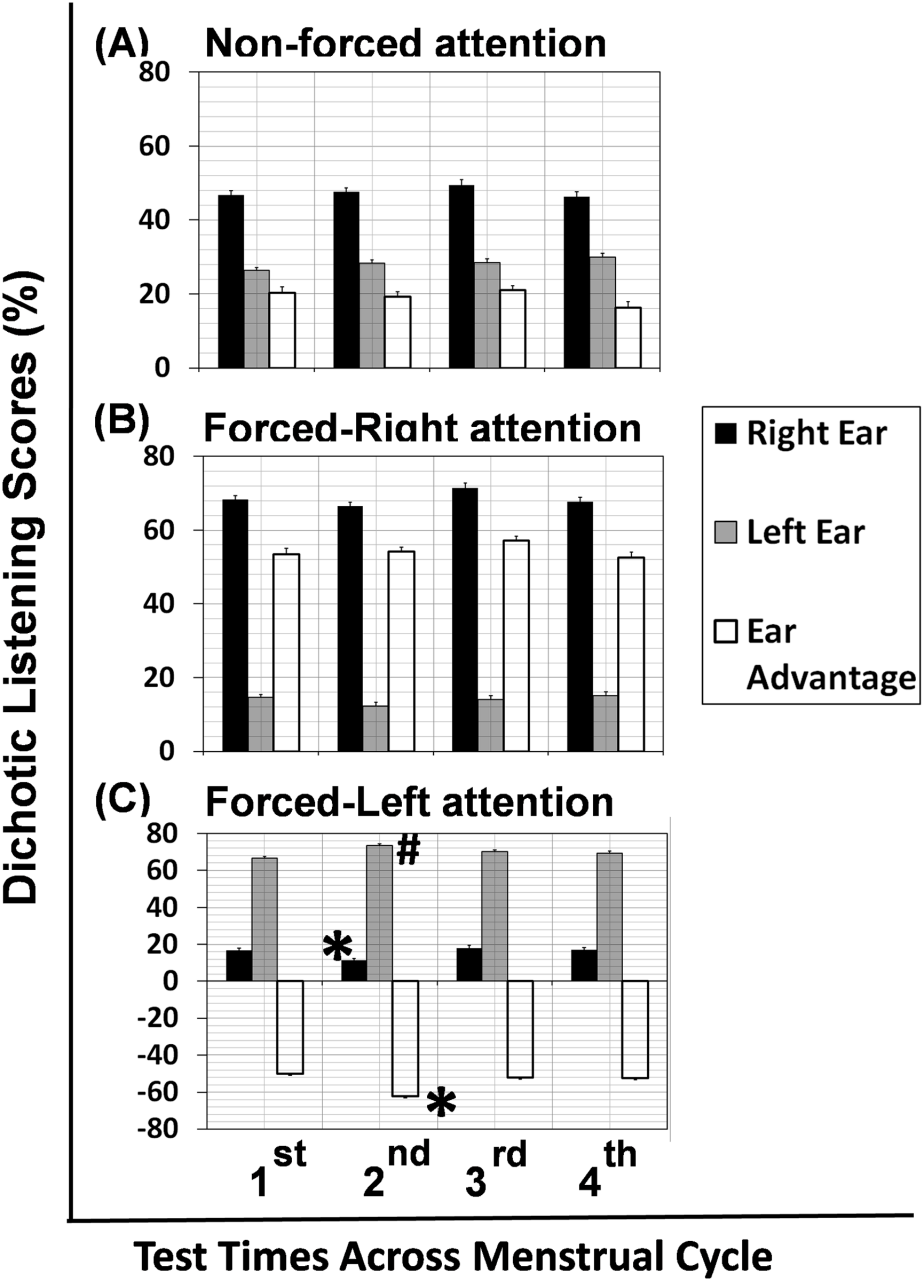
Ear scores and ear advantage across the menstrual cycle in different attention conditions. Error bars are standard errors. The symbol * shows the score at the 2^nd^ test time to be significantly different from that at the other three test times, while the symbol # indicates the score at the 2^nd^ test time to be significantly different from that at the 1^st^ test time.

Fig 2 shows the mean ear scores for the right ear (dark bars), the left ear (grey bars), and ear advantage (unfilled bars) across the menstrual cycle for the non-forced (NF), forced-right (FR) and forced-left (FL) attention conditions. For the NF attention, the right-ear score is approximately 45% across the test times while the left-ear score is around 30%, resulting in a positive ear advantage or a right-ear advantage (REA). For the FR attention, the right-ear score increases to about 70% while the left-ear score drops to 15%. The REA rises to approximately 50%. For the FL attention, the right-ear score drops to less than 20% across the menstrual cycle while the left-ear score increases to about 70%, reversing the ear advantage to a negative value or a left-ear advantage (LEA). The variation of ear scores at each test time is small (<2% in standard error) for each ear across the three listening conditions.

The global 3x2x4 repeated-measures ANOVA was performed on the ear scores with the attention condition (NF, FR, FL), ear (right, left) and test time (1^st^, 2^nd^, 3^rd^, 4^th^) as the within-subjects effects. The results show that the main effects are all significant: attention condition (F(2,38) = 39.57, *p*< 0.001, η^2^ = 0.70, power = 1.0), ear (F(1,19) = 130.82, *p*< 0.001, η^2^ = 0.87, power = 1.0), and test time (F(3,57) = 4.35, *p* = 0.008, η^2^ = 0.19, power = 0.9). The interaction effect of attention condition x ear x test time is also significant, F(6,114) = 3.57, *p* = 0.002, η^2^ = 0.16, power = 0.9. As the effect of ear is significant, the right ear performs differently from the left ear across the attention conditions and the test times. The results of post-hoc analysis to examine the effect of test time on ear score (and ear advantage) at each attention condition are tabulated in Table 2. The effect of test time is significant only for the forced-left attention condition (Table 2). Under the forced-left attention, the right-ear score at the 2^nd^ test time is significantly lower than that at the other three test times, while the left-ear score at the 2^nd^ test time is significantly higher than that at the 1^st^ test time. Consequently, the ear advantage at the 2^nd^ test time is significantly lower than that at the other three test times.

**Table 2:** Results of post-hoc analyses for the effect of menstrual cycle on ear score and ear advantage.

Attention Condition	Ear Score	1-way ANOVA					Significant difference among 4 test times (1^st^, 2^nd^, 3^rd^, 4^th^)? ^a^
		Effect	F(3,57)	*p*	η^2^	power	
Non- Forced	Right	Test time	1.60	0.19	0.07	0.4	None
	Left		2.76	0.06	0.12	0.6	None
	EA		2.36	0.07	0.11	0.5	None
Forced- Right	Right	Test time	2.12	0.10	0.10	0.5	None
	Left		2.78	0.05	0.12	0.3	None
	EA		1.66	0.10	0.08	0.4	None
Forced- Left	Right	Test time	7.53	<0.001	0.28	0.98	2^nd^>1^st^, 3^rd^, 4^th^
	Left		3.57	0.02	0.16	0.76	2^nd^>1^st^
	EA		5.97	0.001	0.28	0.94	2^nd^>1^st^, 3^rd^, 4^th^

EA: ear advantage (right score—left score).

^a^ Multiple comparisons, significance level <0.0125.

### Relation between central conduction time and ear advantage

The relation between the ABR measurements (wave latency, interpeak conduction time) and the ear scores in dichotic listening (under three different attention conditions) is first examined for each ear at each test time. Those pairs that are significantly correlated, *p*<0.05, are tabulated in Table 3. For either ear, the correlation only exists when the dichotic listening task is conducted under the non-forced attention. The correlation is positive at the 1^st^ test time but negative at the 2^nd^ test time. Specifically, the ear score in dichotic listening can relate to the wave latency at peak V, the conduction times at interpeak III-V and interpeak I-V for the right ear, but only the wave latency at peak I for the left ear. Furthermore, the interaural difference in ABR measurements is tested for any relation with the interaural difference in the dichotic listening test (i.e., the ear advantage). The results are also included in Table 3. The relation between these two interaural differences is identified at the 1^st^ test time for the wave latency at peak V and the conduction time at interpeak I-V when the non-forced attention is applied in dichotic listening.

**Table 3:** Significant correlation between measurements in ABR and ear scores in dichotic listening across the test times.

Ear	Test time	ABR condition	Dichotic listening: attention condition	Correlation coefficient, *r*	Significance level, *p*
Right	1^st^	Peak V latency	Non-forced	0.57	0.009
	1^st^	Interpeak III-V conduction time	Non-forced	0.52	0.02
	1^st^	Interpeak I-V conduction time	Non-forced	0.65	0.002
	2^nd^	Peak V latency	Non-forced	-0.55	0.01
	2^nd^	Interpeak III-V conduction time	Non-forced	-0.45	0.04
	2^nd^	Interpeak I-V conduction time	Non-forced	-0.48	0.03
Left	2^nd^	Peak I latency	Non-forced	-0.51	0.02
Right—Left	1^st^	Peak V latency	Non-forced	0.47	0.03
	1^st^	Interpeak I-V conduction time	Non-forced	0.45	0.04

Finally, the relation between the change in the central conduction time (ΔCCT) for the interpeaks I-III, III-V, I-V and the change in the ear advantage (ΔEA) is examined across two test times. The change in the central conduction time is defined either by the sum or by the average of ΔCCT in the right ear and ΔCCT in the left ear. When the sum of the ΔCCT in both ears is used, no relation is found between ΔCCT and ΔEA, *p*>0.05. However, when the average of ΔCCT across ears is used, ΔCCT measured with the interpeak I-V is significantly correlated with ΔEA at two test conditions. These two test conditions are the non-forced attention in dichotic listening at the 2^nd^ test time relative to the 1^st^ test time (r = 0.58, *p* = 0.007), and the forced-left attention at the 2^nd^ test time relative to the 4^th^ test time (r = 0.49, *p* = 0.02). Correlations at other test conditions are not significant, *p*>0.05.

**Fig 3:**
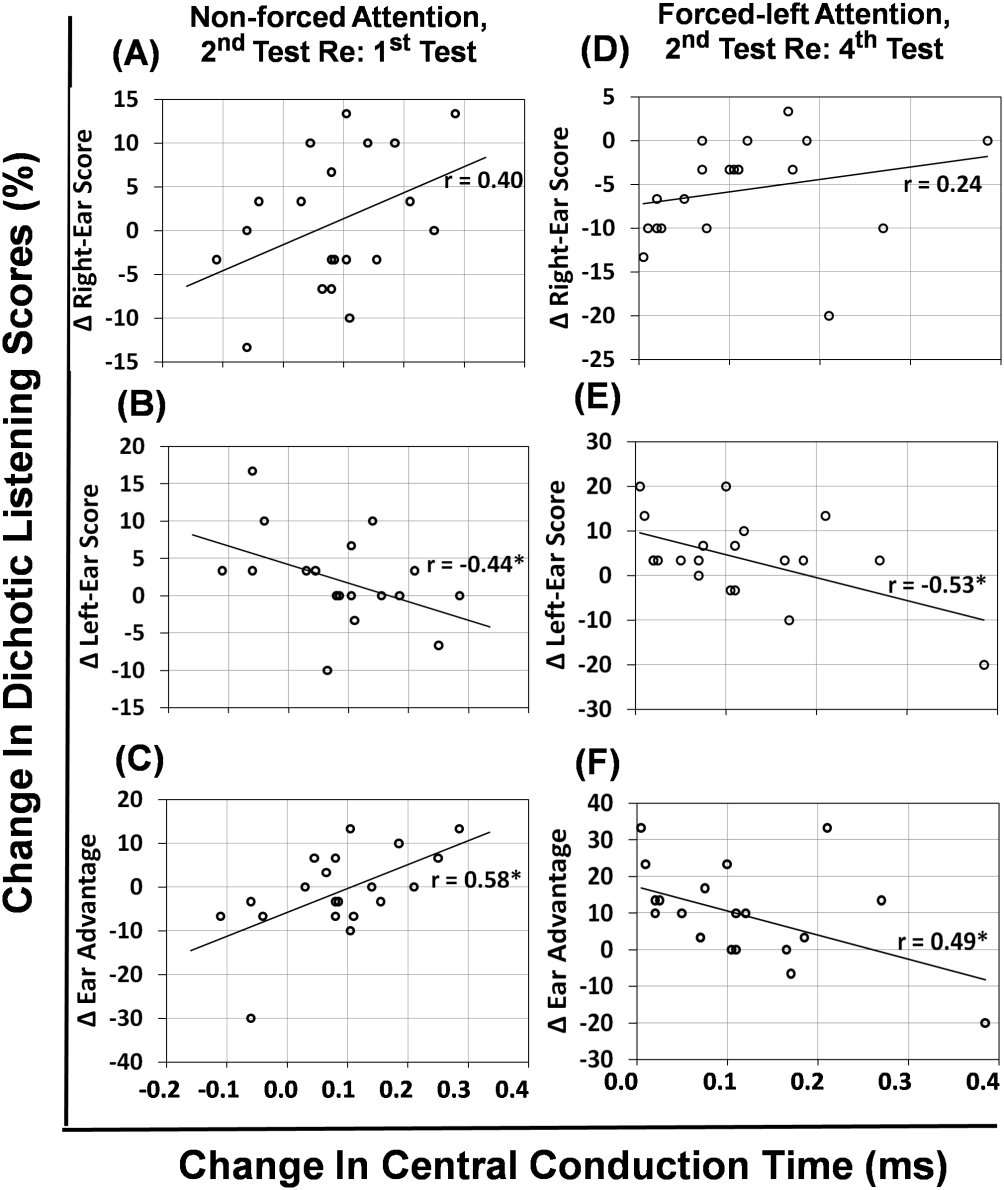
Correlation between the change in dichotic-listening scores and the change in central conduction times. The symbol * indicates that the Pearson’s correlation coefficient r is significant, *p*<0.05.

Fig 3 is a scatterplot to show these two test conditions where ΔCCT is significantly correlated with ΔEA (Fig 3C and 3F). In Fig 3, the change in the right-ear score (Fig 3A and 3D) and the change in left-ear score (Fig 3B and 3E) against ΔCCT are also included. Overall, the change in the right-ear score is elevated as ΔCCT is increased but the correlation is insignificant, *p*>0.05. However, the change in the left-ear score is decreased as ΔCCT is increased across two test times, and the correlation is significant, *p*<0.05. When the change in the left-ear score is subtracted from the change in the right-ear score, the resultant ΔEA is increased at the 2^nd^ test time relative to the 1^st^ test time in the non-forced attention condition, while the resultant ΔEA is decreased at the 2^nd^ test time relative to the 4^th^ test time in the forced-left attention condition.

## Discussion

The primary goal of this paper was to examine if there was any relation between the central conduction time measured in the auditory brainstem response (ABR) and the ear advantage assessed in the dichotic listening task at four test times across the menstrual cycle. To achieve this goal, the respective effects of the menstrual cycle (i.e., test time) on the central conduction time and the ear advantage in dichotic listening were investigated first.

The results of the performance in ABR indicate the effect of test time across the menstrual cycle is more remarkable on the left ear than the right ear (Fig 1). For example, the wave latency at peak V is higher at the 2^nd^ test time than the other test times for both ears but significant difference is only identified for the left ear. However, the results with average ABR across ears show that the wave latency at peak V is significantly lengthened at the 2^nd^ test time relative to the 1^st^, 3^rd^ and 4^th^ test times, but it is not true at peak I and peak III (Fig 1). Consequently, the interpeak conduction times of interpeaks I-V and III-V are also prolonged at the 2^nd^ test time, as the wave latencies at peak I and peak III remain unchanged across the menstrual cycle. Moreover, the power (sensitivity) of test on the central conduction time at the interpeak I-V is high (0.98) (Table 1), suggesting a high likelihood of finding an observable delay in the central conduction time at the 2^nd^ test time during the menstrual cycle. Overall, the current finding demonstrates that the central (interpeak) conduction time depends on the time across the menstrual cycle.

The results of dichotic listening indicate that the ear advantage does not change across different times of the menstrual cycle under the non-forced attention (Fig 2A). This finding suggests that the test time could not produce a significant effect on language lateralization. However, Hodgetts et al. [25] was able to show a significant reduced language lateralization under the non-forced attention by comparing the laterality quotients between the group of women with high estradiol and another group with low estradiol; the grouping was based on a split score 3.4 pg/ml in estradiol. Their findings [25] may explain the current finding of no change in language lateralization (non-forced attention) as the sex hormone level of participants was not required to be higher or lower than a specific value at a particular test time.

When the dichotic listening task is conducted under the forced-left attention, the current study shows that the left-ear score has increased while the right-ear score has decreased at the 2^nd^ test time relative to the other test times, causing a significant increase in the left-ear advantage (Fig 2C). The power (sensitivity) of test on ear advantage is high, 0.94 (Table 2), indicating a high likelihood of finding an observable change in the left-ear advantage at the 2^nd^ test time. On the other hand, no significant shift in the ear advantage is identified at different test times across the menstrual cycle under the forced-right attention (Fig 2B). Therefore, the current finding supports the notion that the ear advantage in dichotic listening under the forced-left attention depends on the time across the menstrual cycle [2].

It is generally accepted that the conduction speed in the nervous system has adaptive value and usually the faster the better in functional performance [33]. This may imply that a decelerated conduction speed (i.e., an increased interpeak conduction time in ABR) may reduce the speech recognition score in dichotic listening when the stimulus travels along the primary (contralateral) auditory pathway to the speech processing areas in the brain. To verify this hypothesis, the relation between the central conduction time measured in ABR and the ear score in dichotic listening is examined at each test time and across two test times.

At each test time, the results for the right ear show that the ear score under the non-forced attention correlates significantly with the central conduction time measured with the interpeaks III-V and I-V at the 1^st^ and 2^nd^ test times (Table 3). The correlation trend is positive at the 1^st^ test time but it becomes negative later at the 2^nd^ test time. Therefore, under the non-forced attention in dichotic listening and at the 1^st^ test time (i.e., 3 to 4 days after the beginning of menses), it seems the left-hemispheric advantage is favorable for listeners with a slower conduction speed (i.e., a longer conduction time) as their right-ear score in dichotic listening is higher. However, at the 2^nd^ test time (i.e., 6 days later), the effect of the menstrual cycle on the central conduction speed may override the left-hemispheric advantage. Thus, for listeners with slower conduction speed their right-ear score in dichotic listening is decreased at the 2^nd^ test time. For the two forced-attention conditions, the selective attention and the cognitive control [2] may complicate the relation between the central conduction time and the ear score in dichotic listening at each test time, and therefore significant correlation is not identified at the right ear. For the left ear, there is no relation between the central conductive time and the ear score in dichotic listening under all attention conditions. This finding may suggest that the left-ear score does not rely on the central conduction speed alone. In addition, under the non-forced attention in dichotic listening, the left-ear score also depends on the performance of the right-ear which is the dominant ear in dichotic listening. When tested under the forced-attention conditions, the performance at the left ear may be complicated with the selective attention to stimuli and cognitive control to direct attention. Because of the significant correlation at the right ear between the central conduction time and the ear score under the non-forced attention, the interaural difference in central conduction time measured with interpeak I-V can also correlate significantly with the ear advantage in dichotic listening at the 1^st^ test time (Table 3). Overall, the interaural difference in central conduction time can correlate to the ear advantage at the beginning of the menstrual cycle.

Between two test times, the change in central conduction time (average across two ears) correlates significantly with the change in ear advantage in dichotic listening only at the 2^nd^ test time referenced to the 1^st^ or 4^th^ test times (Fig 3C and 3F). When the 2^nd^ test time is referenced to the 1^st^ test time under the non-forced attention in dichotic listening, Fig 3A shows a rising but insignificant trend in the right-ear score. This may indicate that the left-hemispheric advantage for the right ear is still dominant in dichotic listening. On the other hand, the change in the central conduction time is significantly correlated to the change in the left-ear score, where slower central conduction speed causes poorer left-ear score (Fig 3B). Interestingly, the resultant change in ear advantage is significantly correlated with the change in central conduction speed at an increasing trend (Fig 3C). Similarly, when the 2^nd^ test time is referenced to the 4^th^ test time under the forced-left attention, Fig 3D shows no significant association between the change in the right-ear score and the change in central conduction speed. The decrease in the central conduction speed also causes the left-ear score to drop and the correlation is significant (Fig 3E). Consequently, a significant decreasing trend in the ear advantage exists (Fig 3F). The processing of speech signal at this forced-left attention is complex. It may involve the reduction of the central conduction speed across two test times [4] and the change in brain activities at the prefrontal cortex when the levels of sex hormones fluctuate [8, 34]. In general, the change in central conduction time across two test times can correlate to the change in ear advantage of dichotic listening.

Although the current research with four assigned test times (5 to 7 days apart) can demonstrate the relation between the change in central conduction time and the change in ear advantage under the overall influence of the menstrual cycle, it is still unknown what roles that individual sex hormones have played. The current paper can therefore be considered as a preliminary study, and future experiments with test days defined by hormone assays may help us understand the interaction among central conduction time, ear advantage, and sex hormones.

## Conclusions

In line with the previous researches in literature, the current study has verified the effects of the test time across the menstrual cycle on the central conduction time measured in auditory brainstem response and on the ear score in dichotic listening. Specifically, the present study is able to demonstrate that the test time can affect both the central conduction time and the ear advantage simultaneously. Moreover, the interaural difference in central conduction time correlates with the ear advantage (non-forced attention) at the beginning of the menstrual cycle. The change in the central conduction time between two test times can also correlate significantly with the change in ear advantage under the non-forced and forced-left attention. Conclusively, the central conduction time depends on the time during the menstrual cycle, which in turn may affect the performance in dichotic listening, but the underlying mechanisms need further investigation.

## Supporting information

S1 Fig ABR wave latency and interpeak conduction time across the menstrual cycle. (PDF)

S2 Fig Ear scores and ear advantage across the menstrual cycle in different attention conditions. (PDF)

S3 Fig Correlation between the change in dichotic-listening scores and the change in central conduction times. (PDF)
