# Supplementary material for: Central conduction time in auditory brainstem response and ear advantage in dichotic listening across menstrual cycle
Source: PLoS One. 2017 Nov 9;12(11):e0187672. doi: 10.1371/journal.pone.0187672 (PMC5679549; doi:10.1371/journal.pone.0187672)
Supplement: S1 Fig — (PDF) [file pone.0187672.s001.pdf]

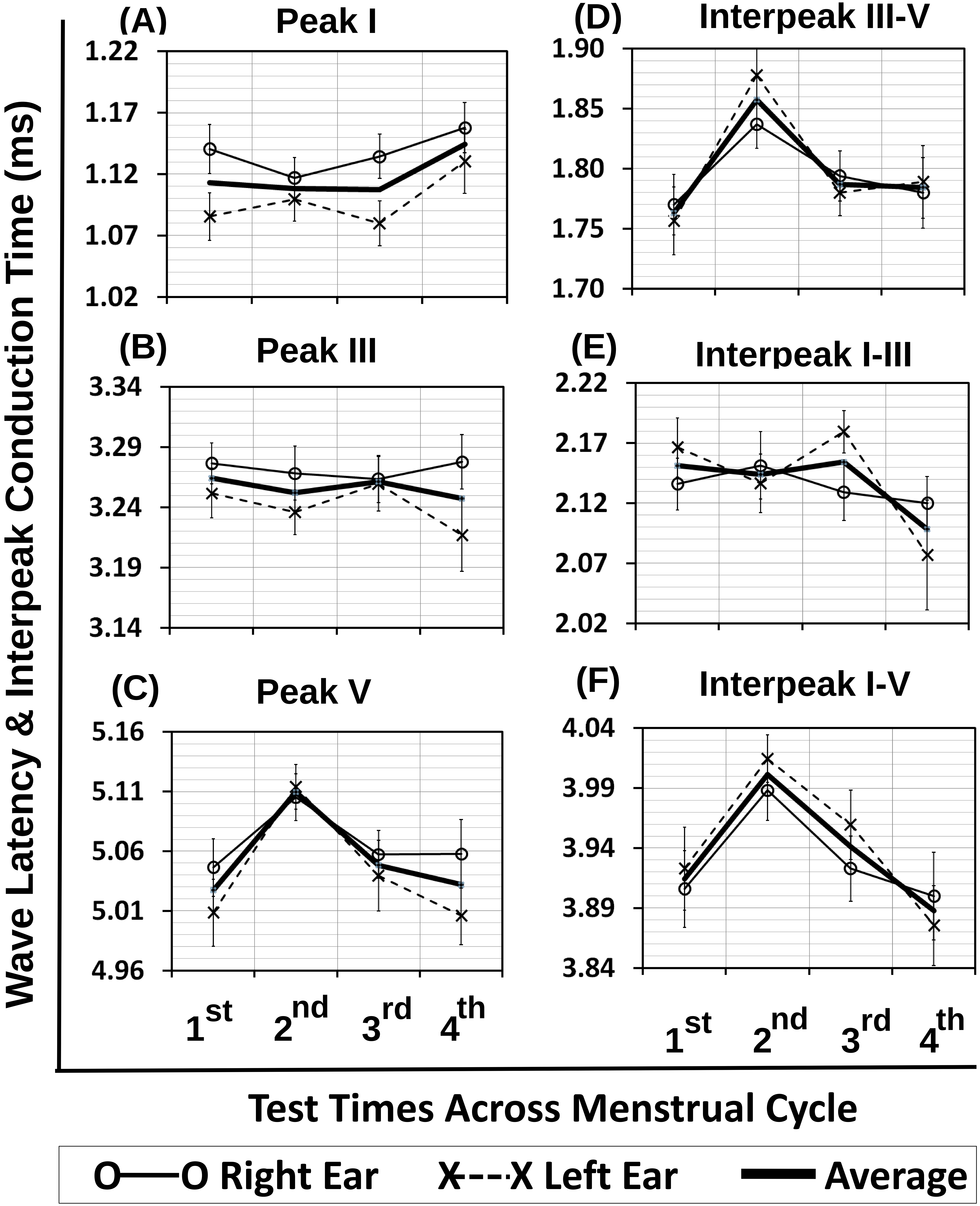

(A) Peak I
(B) Peak III
(C) Peak V
(D) Interpeak III-V
(E) Interpeak I-III
(F) Interpeak I-V
1.22
1.17
1.12
1.07
1.02
3.34
3.29
3.24
3.19
3.14
5.16
5.11
5.06
5.01
4.96
1.90
1.85
1.80
1.75
1.70
2.22
2.17
2.12
2.07
2.02
4.04
3.99
3.94
3.89
3.84
1st 2nd 3rd 4th
Wave Latency & Interpeak Conduction Time (ms)
Test Times Across Menstrual Cycle
O——O Right Ear
X---X Left Ear
Average
