# Supplementary figures and images for: Central conduction time in auditory brainstem response and ear advantage in dichotic listening across menstrual cycle
Source: PLoS One. 2017 Nov 9;12(11):e0187672. doi: 10.1371/journal.pone.0187672 (PMC5679549; doi:10.1371/journal.pone.0187672)

Dichotic Listening Scores (%)

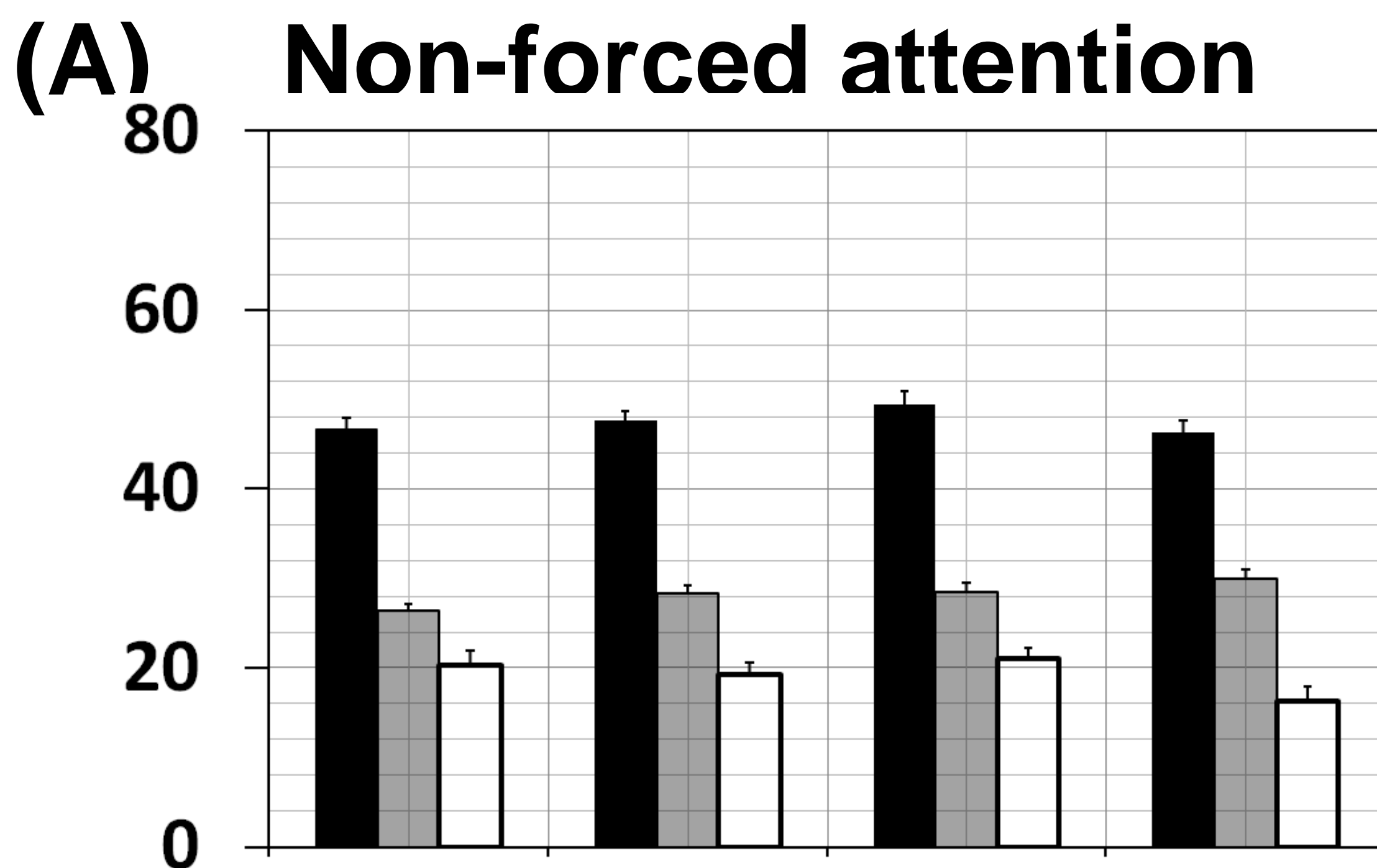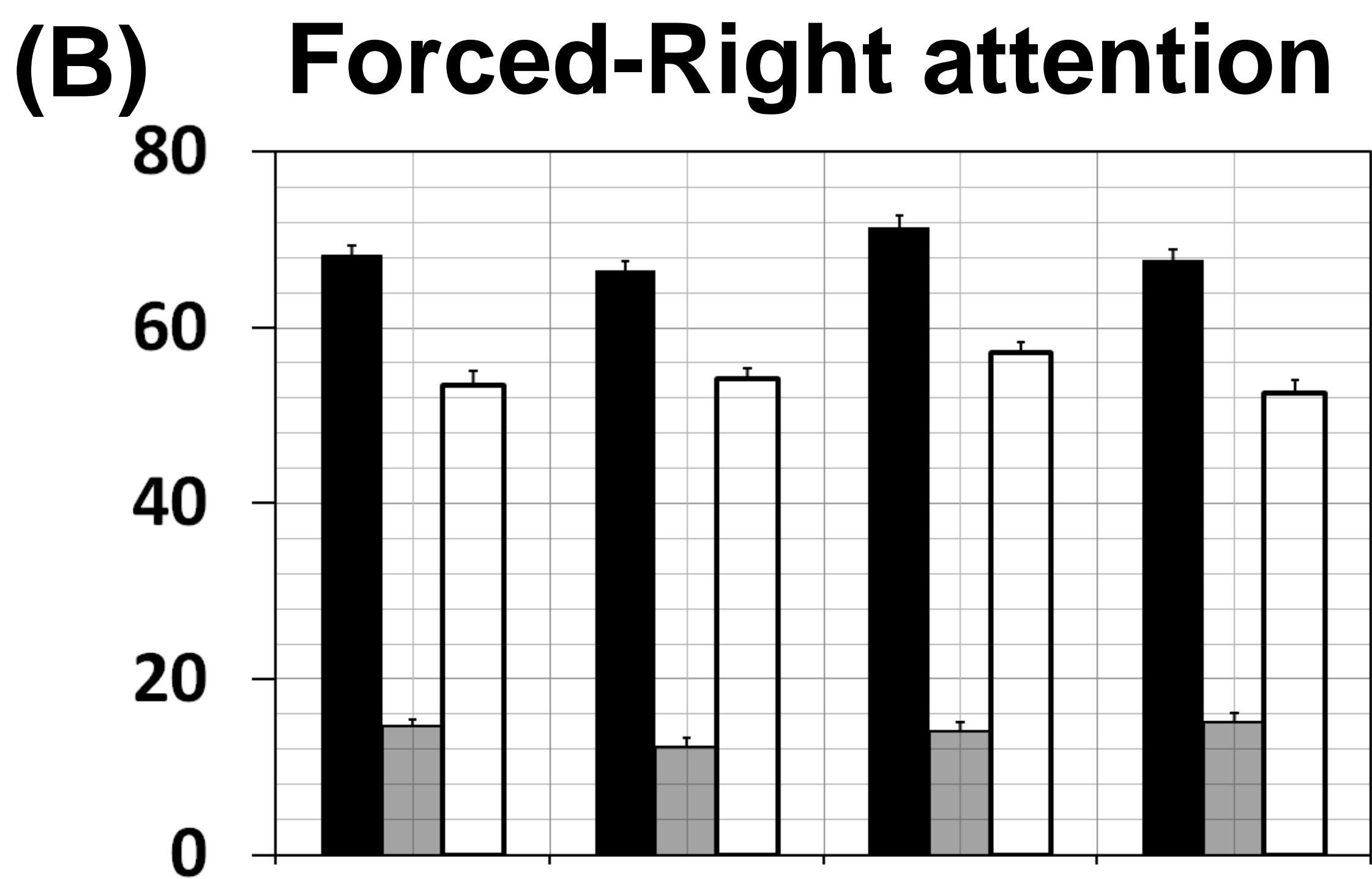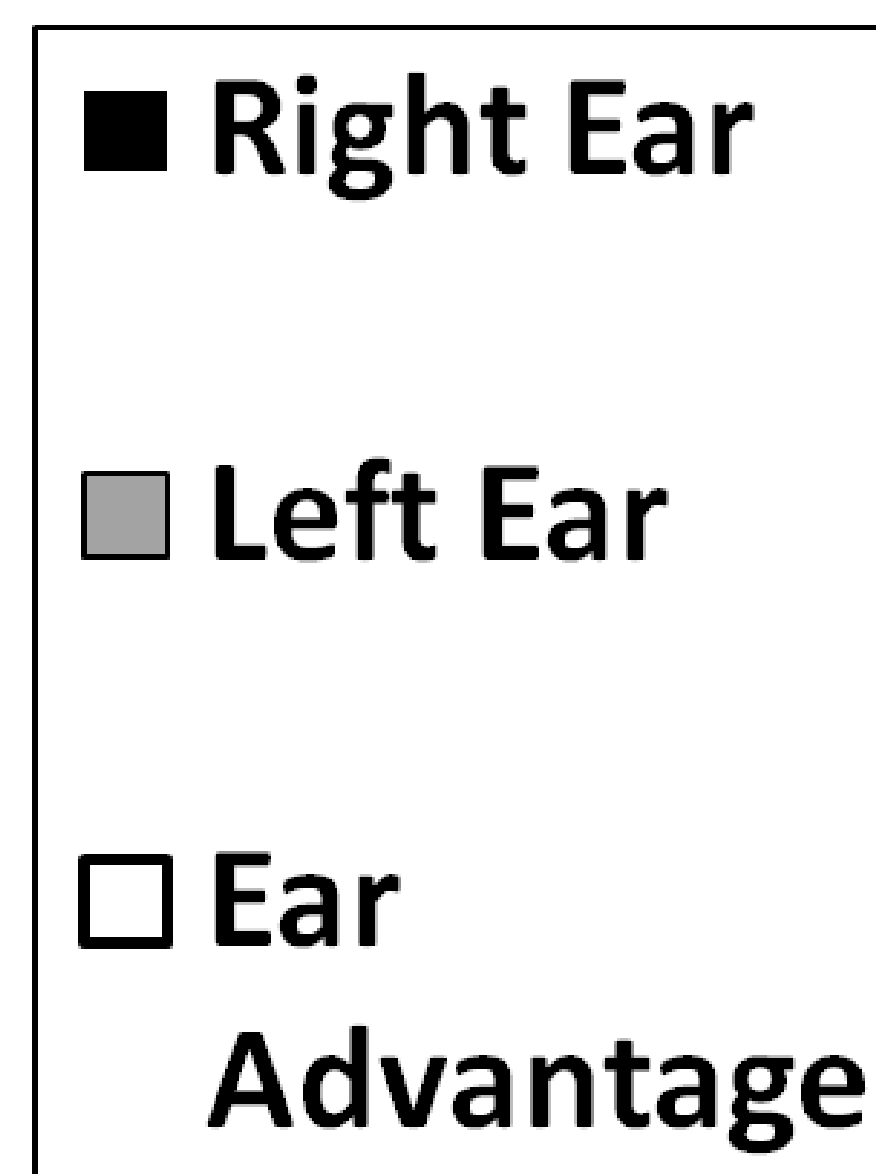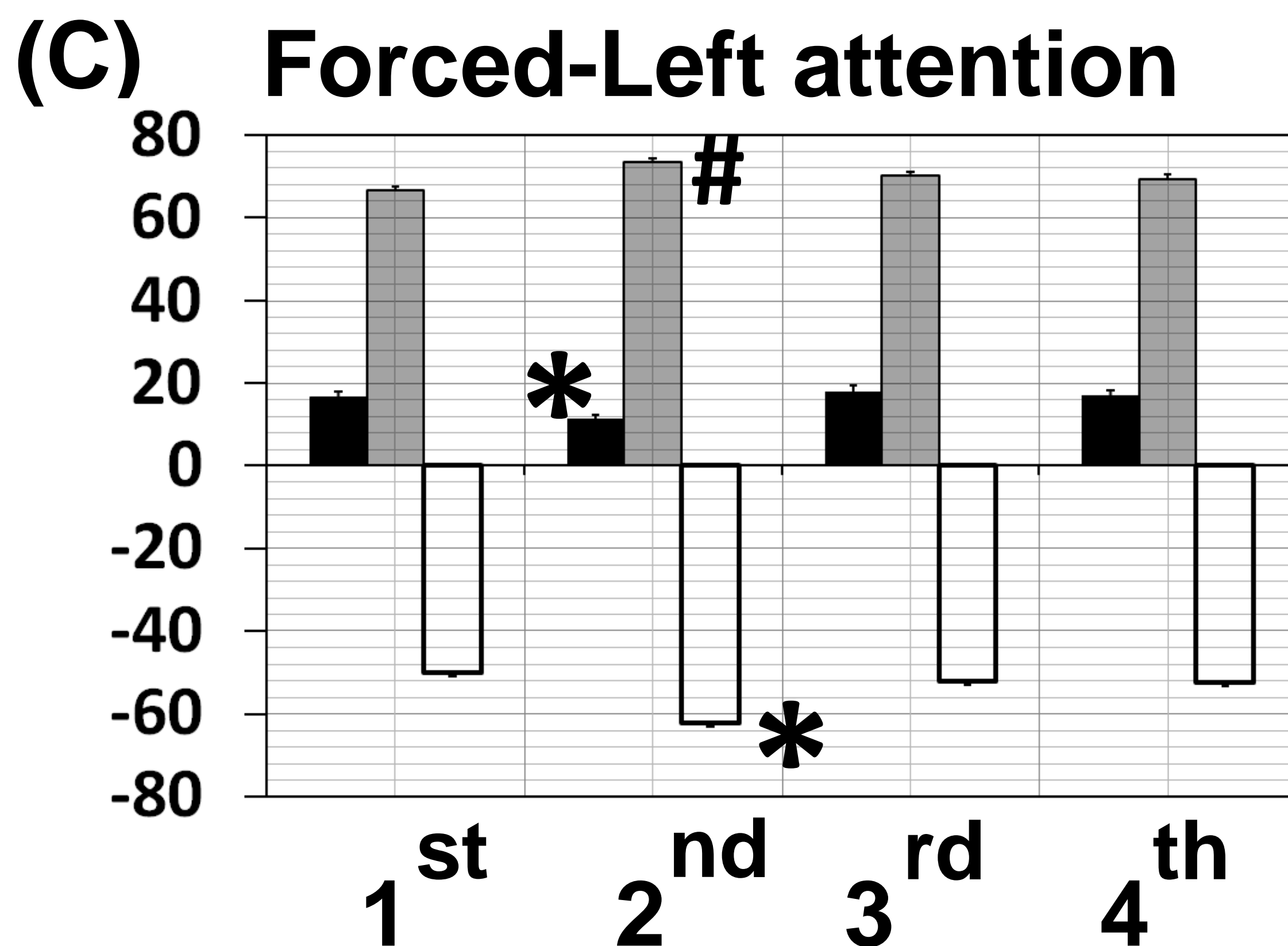

Test Times Across Menstrual Cycle

Supplement: S2 Fig — (PDF) [file pone.0187672.s002.pdf]
