# Supplementary material for: Central conduction time in auditory brainstem response and ear advantage in dichotic listening across menstrual cycle
Source: PLoS One. 2017 Nov 9;12(11):e0187672. doi: 10.1371/journal.pone.0187672 (PMC5679549; doi:10.1371/journal.pone.0187672)
Supplement: S3 Fig — (PDF) [file pone.0187672.s003.pdf]

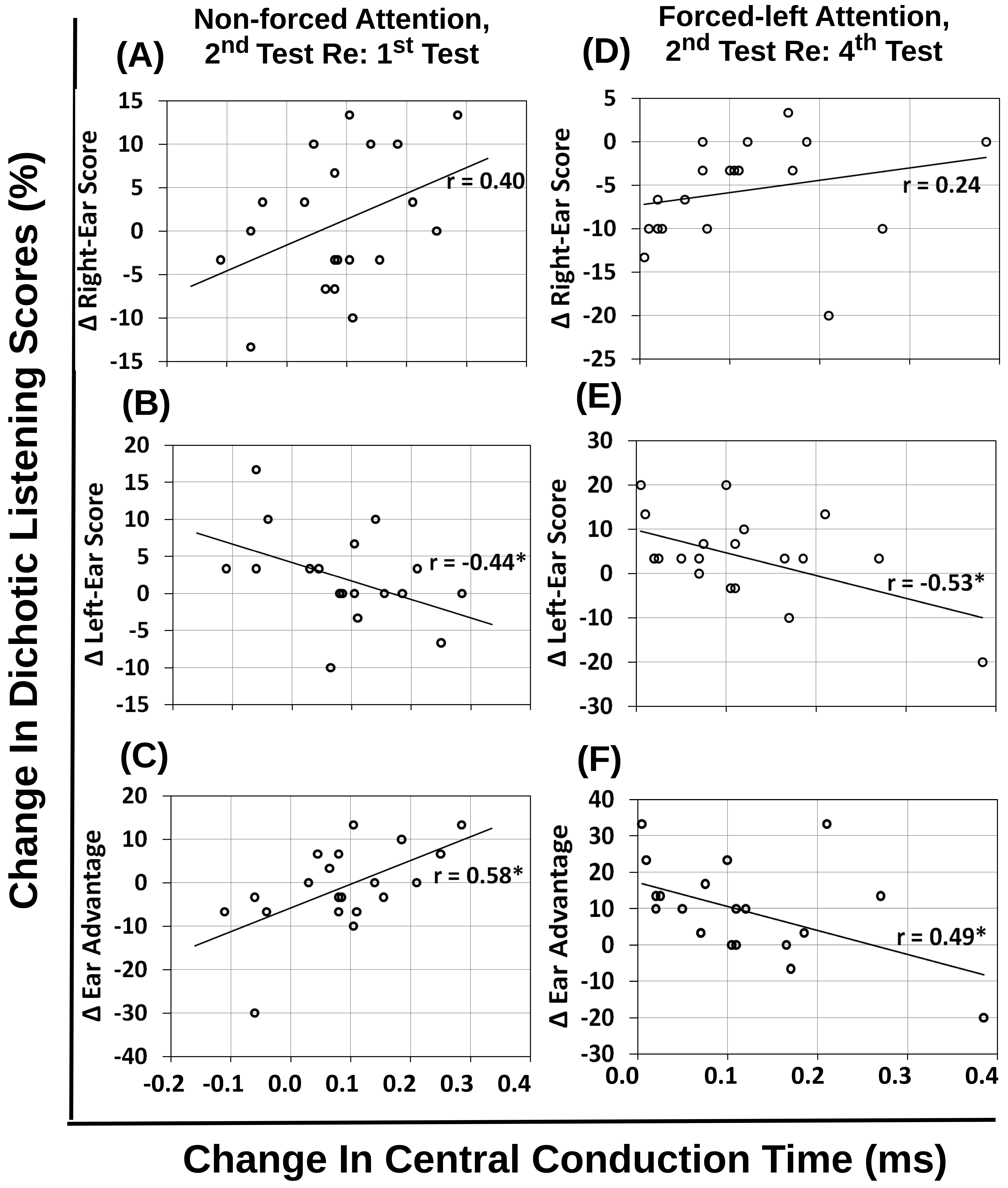

Non-forced Attention,
2nd Test Re: 1st Test
Forced-left Attention,
2nd Test Re: 4th Test
(A)
(B)
(C)
(D)
(E)
(F)
Δ Right-Ear Score
Δ Left-Ear Score
Δ Ear Advantage
r = 0.40
r = -0.44*
r = 0.58*
r = 0.24
r = -0.53*
r = 0.49*
Change In Dichotic Listening Scores (%)
Change In Central Conduction Time (ms)
